# Identifying trajectories of joint space width loss among previously injured knees: Data from the Osteoarthritis Initiative

**DOI:** 10.1371/journal.pone.0325822

**Published:** 2025-06-30

**Authors:** Mary Catherine C. Minnig, Liubov Arbeeva, Jennifer L. Lund, Stephen W. Marshall, Daniel B. Nissman, Amanda E. Nelson, Yvonne M. Golightly

**Affiliations:** 1 Department of Epidemiology, Gillings School of Global Public Health, University of North Carolina at Chapel Hill, Chapel Hill, North Carolina, United States of America; 2 Thurston Arthritis Research Center, University of North Carolina at Chapel Hill, Chapel Hill, North Carolina, United States of America; 3 Department of Radiology, University of North Carolina at Chapel Hill School of Medicine, Chapel Hill, North Carolina, United States of America; 4 College of Allied Health Professions, University of Nebraska Medical Center, Omaha, Nebraska, United States of America; UMC Utrecht: Universitair Medisch Centrum Utrecht, NETHERLANDS, KINGDOM OF THE

## Abstract

**Objectives:**

To identify trajectories of joint space width loss, a proxy measure of tibiofemoral cartilage loss, among previously injured knees. To describe the relationship of trajectory groups with sociodemographic and clinical risk factors.

**Methods:**

Using data from the Osteoarthritis Initiative, we identified right knees with a history of injury. We used group-based trajectory modeling to identify trajectories of joint space width loss over 96-months. Once trajectories were identified, we compared baseline statistics of key risk factors across trajectory groups.

**Results:**

Our primary cohort included 772 previously injured right knees. We also analyzed a subset of 251 more recently injured right knees. Across each cohort, we identified three distinct trajectories for men and women separately, differentiated by low, medium, and high baseline joint space width. Rates of JSW loss were similar between trajectories. Those assigned to the high baseline JSW trajectory were younger at study baseline than those assigned to other two trajectories. Among women assigned to the low baseline JSW group, mean age at the time of knee injury was older than the other two trajectories. Among both men and women, the proportion of knees that had undergone a surgery or arthroscopy was highest in the low baseline JSW group.

**Conclusions:**

Among knees with a history of injury, thinner JSW may be associated with knee surgical history and older age. Moving forward, exploring additional risk factors for OA development among previously injured knees may provide new opportunities to target treatments towards those at the greatest risk for the disease.

## Introduction

Knee osteoarthritis (OA) is a chronic and progressive disease associated with decreased physical function and quality of life [1,2]. OA is currently incurable—treatments options are limited to symptom management [3,4] or total knee arthroplasty (TKA) for end-stage disease [5,6]. No disease-modifying OA drugs (DMOADs) are approved for use [7–9].

Knee OA poses a challenge to DMOAD development due to its high variability of progression and physical indicators. Classifying knee OA into distinct disease phenotypes (groupings distinguishable by various traits) [10–12] may lead to greater development success [8]. Post-traumatic OA (PTOA) is a phenotype characterized by prior injury. According to a 2015 meta-analysis, the odds of knee OA among individuals with a previous knee injury are nearly three times higher compared to uninjured individuals [13]. Additionally, PTOA accounts for roughly 12% of symptomatic OA cases [14,15]. Potentially, individuals at risk for PTOA may be prime participants for DMOAD clinical trials. However, up to roughly 60% of individuals with knee injury history may not develop OA [16]. Determining factors that distinguish those who develop PTOA from those who do not would improve DMOAD clinical trial inclusion criteria and overall DMOAD development.

In this study, we sought to explore longitudinal OA outcomes, defined by changes in joint space width (JSW) measurements (a proxy measure of tibiofemoral cartilage loss), among high-risk knees with prior injury. We used group-based trajectory modeling (GBTM) methods to identify JSW loss trajectories over 96-months. We also sought to describe the distributions of social and clinical characteristics among trajectory groups.

## Methods

### Study sample

This study used a subset of the National Institutes of Health (NIH)-sponsored Osteoarthritis Initiative (OAI) [17], which is publicly available online at https://nda.nih.gov/oai. All OAI is fully anonymized, and all enrolled participants provided informed consent, including written consent at start of each follow-up visit. The OAI is a longitudinal, multicenter, and prospective observational study that was started with the goal of investigating the natural progression of symptomatic OA. The OAI includes 4,796 participants that were recruited between 2004–2006 and invited to annual follow-up visits for up to 8 years. Specific details of the OAI study design have been published previously [18].

**Fig 1:**
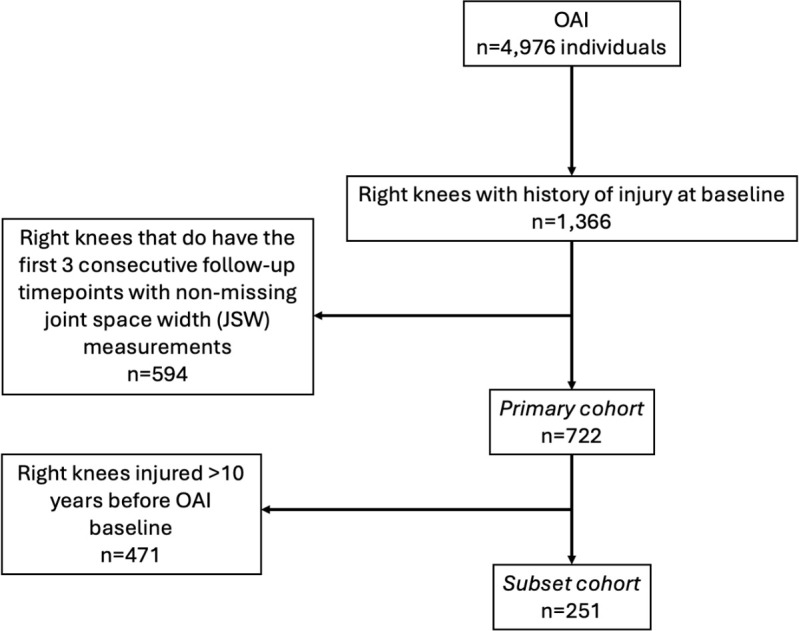
Study enrollment flowchart.

Fig 1 details inclusion criteria for this analysis. To ensure independence between observations, we assessed one knee (the right knee) per participant for inclusion in this analysis. At OAI baseline, we included right knees with history of injury (n = 1366). Injury history was defined through self-report of whether a participant had experienced a knee injury that limited their ability to walk for at least two days. The *primary cohort* was then restricted to knees that had JSW outcome measurements from at least the first three consecutive follow-up timepoints (n = 722). Knees that underwent total knee replacement during follow-up were censored from the timepoint at which replacement was reported. The *subset cohort* was further restricted to knees with “recent” injuries that occurred ≤ 10 years of OAI baseline (n = 251).

### Outcome

We defined OA trajectories by JSW loss over time. Quantitative radiographic JSW measures joint space narrowing in millimeters (mm). Currently, the US Food and Drug Administration recommends that clinical trials investigating potential DMOADs use JSW to determine participant eligibility criteria and serve as the studies’ structural endpoints [8,9]. As part of the OAI imaging protocol, JSW measures were collected at various positions in the joint. ^17^ For this analysis, we used JSW values at the x = 0.250 location in the medial tibiofemoral joint as this measurement location produces the most reliable and responsive gauge of medial tibiofemoral OA progression [19]. JSW measurements were collected at OAI baseline, 12-months, 24-months, 36-months, 48-months, 72-months, and 96-months.

### Sociodemographic and clinical risk factors

Measures collected at baseline included age, age at time of knee injury, race (white and non-white), body mass index (BMI, kg/m^2^), and self-report history of ever having had a right knee surgery or arthroscopy (yes/no).

### Statistical analysis

We used GBTM (PROC TRAJ in SAS Version 9.4) to identify knees that followed similar patterns of JSW loss over 96-months of follow-up [20]. GBTM analyses were conducted separately for men and women because men have on average thicker cartilage in the medial knee joint compared to women, and we anticipated potential differences by sex [21,22].

GBTM uses maximum likelihood functions to fit semiparametric mixture models to longitudinal data. This approach is able to capture sample variability/heterogeneity that cannot be directly observed (i.e., latent), but can be estimated by their effects on observable JSW trajectory. Each observation (i.e., knee) is assigned a probability of belonging to each latent trajectory determined by GBTM [20,23]. We followed established procedures to determine the number of trajectories and their shape (linear or quadratic) [24]. Specifically, the model with the optimal number of trajectory groups was selected based on Bayesian Information Criterion (BIC). Polynomial orders were determined for each trajectory based on their associated statistical significance (p < 0.05). We followed the Guidelines for Reporting on Latent Trajectory Studies (GRoLTS) [25].

We conducted a sensitivity analysis ensure that results were similar between the larger n = 1366 cohort of all injured right knees at OAI baseline and the *primary cohort* of n = 772 right knees used in this analysis (Fig 1, S1 Table, and S2 Table).

Attrition occurred at later follow-up timepoints, affecting missingness in the JSW outcome measure (12.3%, 38.6%, and 37.6% of JSW observations were missing at the 48-month, 72-month, and 96-month follow-ups). Therefore, once the ideal model was selected, we included a dropout statement extension in the PROC TRAJ program [23,26].

Posterior probabilities of group membership were assessed to determine model fit, where probabilities of ≥70% represent good fit [27]. We used descriptive statistics to summarize sociodemographic and clinical features stratified by trajectory group membership. Frequencies of missing JSW observations in the *primary cohort* at the 36-month, 48-month, 72-month, and 96-month follow-ups were stratified by trajectory group to describe patterns of missingness (S1 Fig). Due to the significant amount of missingness in the *primary cohort*, we conducted a supplemental analysis where missing JSW observations at the 36-month, 48-month, 72-month, and 96-month follow-ups were imputed using the last observation carried forward method (S7 Table, S8 Table, and S2 Fig).

To assess how time from injury to OAI enrollment affected trajectory group patterns, we conducted an additional analysis in a subset of knees from the *primary cohort* that experienced an injury ≤10 years of OAI baseline (denoted as the *subset cohort*).

### Ethical approval information

The OAI was overseen by an independent Observational Study Monitoring Board (OSMB) appointed by the National Institute of Arthritis and Musculoskeletal and Skin Diseases (NIAMS) and the National Institutes of Health (NIH) between 2002 and 2014.

## Results

### Primary cohort

#### Description of trajectory groups.

**Fig 2:**
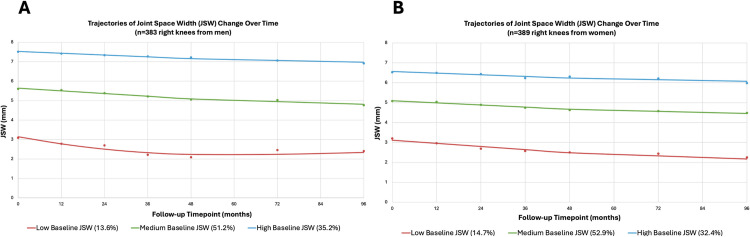
Joint space width (JSW) trajectory groups across 96-months of follow-up among the *primary cohort.* **Panel A** shows trajectories among previously injured right knees from men (n = 383), while **Panel B** shows trajectories among previously injured right knees from women (n = 389). The red trajectories represent the “low baseline JSW” group, the green trajectories represent the “medium baseline JSW” group, and the blue trajectories represent the “high baseline JSW” group.

We chose models with three trajectory groups for both men and women separately (S3 Table and S4 Table). Trajectory patterns over time were similar between biological sex (Fig 2). Rates of JSW loss over follow-up among different trajectory groups were similar, but their intercepts differed based on JSW measurements at OAI baseline. For ease of description, groups are labeled as: “low baseline JSW” (red trajectory), “medium baseline JSW” (green trajectory), and “high baseline JSW” (blue trajectory). Average posterior probabilities for each trajectory group were 97–98% for men and 96–98% for women.

Among men, the largest and smallest proportions of observations belonged to the medium (51.2%) and the low (13.6%) baseline JSW groups, respectively. Trajectory intercepts (baseline JSW measurements) were 3.6 mm (low group), 5.8 mm (medium group), and 7.6 mm (high group). Among women, the largest and smallest proportions of observations also belonged to the medium (52.9%) and the low (14.7%) baseline JSW groups, respectively. Trajectory intercepts were 3.3 mm (low group), 5.2 mm (medium group), and 6.6 mm (high group).

### Cohort description

The cohort consisted of an equal proportion of men and women (Table 1). Age at time of injury was 4–77 years, with roughly 7% of women and 3% of men reporting an injury at ≤10 years of age. Mean age at OAI baseline was younger among the high baseline JSW group for men and women compared to medium and low groups. Among women, mean age at time of injury was older in the low group (47.5 years) compared to the medium and high groups (39.0 and 35.0 years, respectively). Among men, mean age at time of injury did not differ greatly between groups, and the proportion of knees with a surgery or arthroplasty was highest in the low group (73.6% vs. 55.9% and 41.0% in the medium and high groups, respectively). Among women, 38.6% of knees belonging to the low group had a surgery or arthroplasty, compared to 26.0% and 22.7% of those assigned to the medium and high groups, respectively.

**Table 1:** Baseline sociodemographic and clinical characteristics (n = 772) stratified by sex and group membership among the *primary cohort.*

	Men (n = 383)			Women (n = 389)		
	Baseline right knee JSW trajectory group			Baseline right knee JSW trajectory group		
	Low	Medium	High	Low	Medium	High
**Age at OAI baseline,** years, mean ± SD, range	61.0 ± 9.4, 46 - 76	60.4 ± 9.0, 45 - 79	57.6 ± 8.5, 45 - 78	65.1 ± 8.4, 45 - 79	61.1 ± 9.0, 45 - 79	59.2 ± 8.7, 45 - 78
**Age at time of injury** mean ± SD, range	33.8 ± 18.0, 8 - 74	35.2 ± 18.2, 8 - 77	32.0 ± 16.3, 9 - 73	47.5 ± 19.3, 4 - 74	39.0 ± 19.4, 4 - 75	35.0 ± 17.9, 4–74
**Race** n (%) *White* *Non-white*	48 (90.6) 5 (9.4)	177 (90.8) 18 (9.2)	117 (86.7) 18 (13.3)	47 (82.5) 10 (17.5)	160 (78.4) 44 (21.6)	97 (75.8) 31 (24.2)
**BMI**, kg/m^2^, mean ± SD, range	29.0 ± 3.9, 21.5 - 37.9	29.0 ± 4.4, 19.6 - 41.3	29.5 ± 3.9, 21.4 - 40.9	29.8 ± 5.8, 18.6 - 48.7	29.0 ± 5.5, 18.2–44.2	29.6 ± 5.6, 17.6–46.7
**Ever had right knee surgery or arthroscopy** n (%) *Yes* *No*	39 (73.6) 14 (26.4)	86 (55.9) 109 (44.1)	55 (41.0) 79 (59.0)	22 (38.6) 35 (61.4)	53 (26.0) 151 (74.0)	29 (22.7) 99 (77.3)

### Subset cohort

#### Description of trajectory groups.

**Fig 3:**
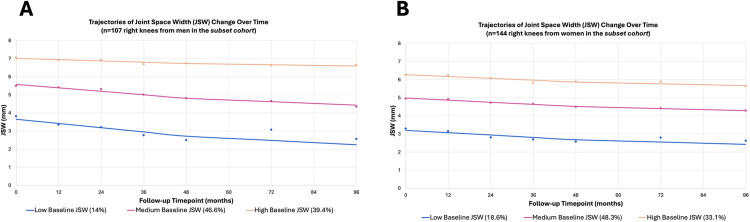
Joint space width (JSW) trajectory groups across 96-months of follow-up among the *subset cohort.* **Panel A** shows trajectories among male right knees that were injured within 10 years before OAI baseline (n = 107), while **Panel B** shows trajectories among previously female knees that were injured within 10 years before OAI baseline (n = 144). The blue trajectories represent the “low baseline JSW” group, the pink trajectories represent the “medium baseline JSW” group and the orange trajectories represent the “high baseline JSW” group.

We chose models with three trajectory groups for both men and women separately in the *subset cohort* (knees injured within 10 years prior to OAI baseline) (S5 Table and S6 Table). Like the *primary cohort,* trajectories were primarily differentiated by JSW measures at OAI baseline (Fig 3). Among men, trajectory intercepts were 7.1 mm (high group), 5.5 mm (middle group), and 3.8 mm (low group). Among women, trajectory intercepts were 6.3 mm (high group), 4.9 mm (medium group), and 3.3 mm (low group). Average posterior probabilities for each trajectory were 96–97% for men and 95–97% for women. The largest proportion of the cohort was assigned to the medium group (46.6% and 48.3% for men and women, respectively), followed by high (39.4% and 33.1%) and low (14.0 and 18.6%) groups. Among women, rates of JSW loss were similar across trajectories. Among men, rate of JSW loss appeared greater for the medium and low groups between OAI baseline and 48-months compared to the high group.

### Cohort description

The cohort was 57% women and age at time of injury ranged from 35 to 77 years (Table 2). Mean age at OAI baseline and mean age at time of injury were lowest among those in the high group. Compared to men, there was a larger difference between the mean age at time of injury for women in the low group (62.1 years) and the medium and high groups (56.3 and 66.7 years, respectively). Among men, the proportion of knees with surgery or arthroscopy was highest in the low group (80% vs. 46.9% and 51.2% in the medium and high groups, respectively). We observed a similar pattern among women, where 37% of knees in the low group had surgery or arthroscopy (vs. 27.5% and 14.6% of those in the medium and high groups).

**Table 2:** Baseline sociodemographic and clinical characteristics (n = 251) stratified by sex and group membership among the *subset cohort.*

	Men (n = 107)			Women (n = 144)		
	Baseline right knee JSW trajectory group			Baseline right knee JSW trajectory group		
	Low	Medium	High	Low	Medium	High
**Age at OAI baseline,** years, mean ± SD, range	61.2 ± 10.3, 46 - 76	60.6 ± 7.9, 47 - 79	58.3 ± 8.9, 45 - 78	65.5 ± 7.2, 55 - 79	60.5 ± 7.2, 55 - 78	60.5 ± 8.7, 47 - 78
**Age at time of injury** mean ± SD, range	56.0 ± 11.4, 41 - 74	55.9 ± 8.5, 40 - 77	53.7 ± 10.4, 35 - 76	62.1 ± 7.0, 48 - 74	56.3 ± 9.5, 39 - 75	56.7 ± 9.1, 42–74
**Race** n (%) *White* *Non-white*	13 (86.7) 2 (13.3)	46 (93.9) 3 (6.1)	37 (86.1) 6 (13.9)	21 (77.8) 6 (22.2)	51 (73.9) 18 (26.1)	36 (75.0) 12 (25.0)
**BMI**, kg/m^2^, mean ± SD, range	29.9 ± 3.2, 26.3 - 37.9	28.5 ± 3.5, 22.1–36.7	29.7 ± 3.9, 23.2–39.5	29.8 ± 5.1, 18.6–39.8	29.3 ± 5.1, 18.6–39.8	29.9 ± 6.0, 19.5–44.8
**Ever had right knee surgery or arthroscopy** n (%) *Yes* *No*	12 (80) 3 (20)	23 (46.9) 26 (53.1)	22 (51.2) 21 (48.8)	10 (37.0) 17 (63.0)	19 (27.5) 50 (72.5)	7 (14.6) 41 (85.4)

## Discussion

In this study of injured knees, we identified three trajectories of JSW loss among men and three trajectories among women. In the *primary cohort*, we did not find that these groups had differing rates of JSW loss. Previous studies have shown a potential link between knee injury and accelerated knee OA development, defined as progression from pre-radiographic disease to advanced-stage disease in less than four years [28,29]. This study has the advantage of a long period of time between joint injury and entry into the OAI parent study. In the *primary cohort*, age at the time of injury ranged from 4 to 77 years, while entry into the OAI occurred between 45–79 years of age. This extended time between injury and trajectory assessment may explain why we do not observe a trajectory characterized by a rapid decrease in JSW in the *primary cohort*—these changes likely occurred closer to the time of injury and prior to OAI entry.

In men only, we observed more rapid JSW loss in the low and medium baseline JSW groups in the *subset cohort*, which included knees that were injured within 10 years of OAI baseline. Current literature does not suggest a relationship between biologic sex and accelerated knee OA [29]. However, future studies investigating the complex interaction between injury, sex, sex hormones, and age are needed to better understand the potential association between sex and varying knee OA progression [29].

The proportion of right knees that had undergone surgery or arthroplasty was highest among those in the low baseline trajectory group for both the *primary* and *subset cohorts*. Surgery following an injury may occur for several reasons. For an anterior cruciate ligament (ACL) injury, surgical ACL reconstruction may be recommended to improve joint stability, minimize secondary injuries to the meniscus, and potentially reduce the risk of OA development [30]. Studies comparing the incidence of OA between individuals with surgical repair versus conservative treatment (i.e., rehabilitation) are limited, however. A systematic review found that long-term OA incidence (with mean follow-up of 11.8 years) following ACL reconstruction was 28–87% compared to 11–73% following conservative management [31]. Another study found no significant difference in the development of OA (9–11 years post-injury) between individuals with and without surgical ACL repair [32]. These studies have typically been limited to ACL injuries and reconstruction with fewer investigations into other types of injuries and surgeries. To date, evidence does not suggest that surgery or conservative treatment significantly reduce the incidence of OA.

Particularly in the *primary cohort,* age at time of injury was inversely associated with baseline JSW measurements, where individuals who were older at baseline were more likely to belong to the low baseline JSW group. This trend was stronger among women; the mean age at OAI baseline among those in the low baseline JSW group was over 12 years older compared to those in the high baseline JSW group. Age at OAI baseline followed a similar pattern, where older individuals were more likely to belong to the low baseline JSW groups compared to the high baseline JSW groups.

Older age is a well-known risk factor for OA development, likely due to changes to the synovium, infrapatellar fat pad, and articular ligaments that may increase joint vulnerability as we age [33]. Age is associated with specific changes to articular cartilage health as well, including decreases in cartilage water content and cell density in its superficial layers [33]. In this study, mean age at the time of knee injury was older for women assigned to the low baseline JSW group. While evidence is limited, a systematic review indicated that older adults may experience poorer physical function following a traumatic injury compared to younger adults and/or their pre-injury health status, indicating a decreased ability to recover from injury with older age [34]. These findings suggest that older women with an injury may experience more rapid cartilage thickness decline than younger women with an injury, although additional studies are needed to investigate this idea further.

Among men in the *primary cohort,* we found a higher proportion of missing JSW observations in the low baseline JSW trajectory group compared to the medium and high groups. To address the effects of missingness on trajectory shapes and patterns, we conducted a supplementary analysis of the *primary cohort* where missing JSW measurements were imputed using the last observation carried forward method. Among men, we observed similar trajectory shapes and patterns in the medium and high baseline JSW trajectories between the non-imputed and imputed cohorts. The low baseline JSW trajectory in the *primary cohort* followed a quadratic shape while the low baseline JSW group in the imputed cohort followed a linear shape with a steeper rate of JSW loss. The last observation carried forward method is limited because it assumes no change in JSW measurements from the last time it was assessed.

We chose to use GBTM for this descriptive analysis because of its ability to explore patterns in longitudinal data and for the ease of interpretability of results. One of GBTM’s strengths is that it allows for flexibility in capturing nonlinear (e.g., quadric, cubic, or spline) trends over time [27]. Future studies on this topic may use other methods for assessing trajectories, such as growth curve modeling (GCM), growth mixture models (GMMs), or latent class analysis [35,36]. As the use of machine and deep learning algorithms continues to expand in rheumatic and musculoskeletal disease research, advanced trajectory modeling techniques, including k-means clustering, would also be useful to group heterogenous data [37].

A strength of this study is the use of a broad definition of knee injury—whether an injury had limited one’s ability to walk for at least two days—as a basis for participant inclusion in analysis. We chose this injury classification because it includes individuals with knee injury who may not have received a formal diagnosis from a healthcare provider. Individuals may choose to abstain from seeking medical care for myriad reasons, including a perceived low need for care, insufficient medical insurance coverage, or due to perceived inconvenience [38,39]. It is possible that limiting inclusion to individuals with a professionally diagnosed injury would lead to a cohort that does not experience the typical barriers to seeking medical care.

### Limitations

The OAI cohort is comprised of middle-aged to older adults who are predominantly white, non-Hispanic and of higher socioeconomic status than the general population; thus, this study may be less generalizable to groups of younger, more active, and diverse individuals. We noted some of the strengths of GBTM earlier, but this method also has several limitations. First, while there are guidelines for selecting models of best fit within GBTM, the decision is somewhat subjective. Model selection is also specific to each cohort, thus limiting generalizability. GBTM assumes that data are missing at random due to its use of full information maximum likelihood estimation, however, some data could be missing for non-random reasons. Finally, GBTM does not allow random effects (i.e., inter-individual variability between subjects within latent trajectories) and imposes the same residual variance over time and over all trajectories [40]. Future studies may use different trajectory analyses, such as growth mixture modeling [41] or generalized additive mixed modeling [42], that allow for variability of these factors within groups.

The amount of missing JSW observations at later timepoints is a limitation of our study, and something commonly reported in longitudinal research. As previously mentioned, we addressed missingness by including a dropout statement to *Proc traj*. Additionally, to explore how missingness may affect trajectory results, we conducted a few supplemental analyses. We examined a histogram of the proportion of missing JSW observations stratified by group membership (S9 Fig) and a supplemental analysis where missing JSW observations were imputed (S7 and S8 Tables, and S10 Fig). We do not observe significant differences between the trajectories identified using the *primary cohort* and those identified the *imputed cohort*.

We also note the time between data collection points (1–2 years in this study) may impact the shape and “smoothness” of the trajectories we identified. Longer intervals between data collection timepoints may inherently miss shorter-term fluctuations in JSW measurements, leading to an underestimation of within-person variation. While we view our broad definition of joint injury used to determine study inclusion criteria as a strength, it is important to recognize that the definition does not provide insight into the severity of the injury. Individuals who experienced a severe injury that led to rapid OA progression and joint replacement surgery may be inherently excluded from the cohort studied in this analysis.

### Research implications

Study results expand our understanding of tibiofemoral cartilage thickness changes after injury. We sought to explore risk factors associated with different trajectories of JSW loss over time and found that knee surgery or arthroplasty as well as older age may be important factors to investigate further. We suspect that future studies using methods able to predict outcomes (including deep learning algorithms) will be an effective addition to the literature on this topic. Ultimately, we believe that these studies will be important to consider when deciding on participant enrollment strategies for DMOAD clinical trials. Improving our understanding of who among a group of high-risk individuals go on to experience JSW thinning will be crucial to redefining DMOAD clinical trial participant inclusion criteria.

## Conclusion

This study suggests distinct patterns of JSW loss among knees with prior injury. Knees with injury and a surgical or arthroscopic procedure may be more likely to have reduced JSW than those with injury alone. Women who were older at the time of a knee injury may be more likely to have reduced JSW compared to women who were younger at the time of injury occurrence. Results indicate that certain sociodemographic and clinical features could be associated with differing OA outcomes longitudinally. Identifying and understanding these trajectories and their risk factors will be important for moving forward in the DMOAD clinical trial space.

## Supporting information

S1 Table Model statistics for sensitivity analysis #1 (men). Censored normal distribution model fitting statistics for group-based trajectory modeling among men (from the n = 1366 knee cohort). Models include time (independent variable) and joint space width (dependent variable).
(DOCX)

S2 Table Model statistics for sensitivity analysis #1 (women). Censored normal distribution model fitting statistics for group-based trajectory modeling among women (from the n = 1366 knee cohort). Models include time (independent variable) and joint space width (dependent variable).
(DOCX)

S3 Table Model statistics for primary analysis (men). Censored normal distribution group-based trajectory model fitting statistics for n = 383 right knees from men in the *primary cohort*. Models include time (independent variable) and joint space width (dependent variable).
(DOCX)

S4 Table Model statistics for primary analysis (women). Censored normal distribution group-based trajectory model fitting statistics for n = 389 right knees from women in the *primary cohort*. Models include time (independent variable) and joint space width (dependent variable).
(DOCX)

S5 Table Model statistics for sensitivity analysis #2 (men). Censored normal distribution group-based trajectory model fitting statistics for n = 107 right knees from men in the *subset cohort*. Models include time (independent variable) and joint space width (dependent variable).
(DOCX)

S6 Table Model statistics for sensitivity analysis #2 (women). Censored normal distribution group-based trajectory model fitting statistics for n = 144 right knees from women in the *subset cohort*. Models include time (independent variable) and joint space width (dependent variable).
(DOCX)

S7 Table Model statistics for sensitivity analysis #3 (men). Censored normal distribution group-based trajectory model fitting statistics for n = 383 right knees from men in the *imputed cohort*. JSW measurements were imputed using the “last observation carried forward” method. Models include time (independent variable) and joint space width (dependent variable).
(DOCX)

S8 Table Model statistics for sensitivity analysis #3 (women). Censored normal distribution group-based trajectory model fitting statistics for n = 389 right knees from women in the *imputed cohort*. JSW measurements were imputed using the “last observation carried forward” method. Models include time (independent variable) and joint space width (dependent variable).
(DOCX)

S1 Fig Distribution of missing JSW observations across trajectory groups. Frequencies of missing JSW observations at the 36-month, 48-month, 72-month, and 96-month follow-ups stratified by assigned trajectory groups among the *primary cohort*. The left-hand distribution includes knees from men, while the right-hand distribution includes knees from women.
(PNG)

S9 Table CHecklist for Statistical Assessment for Medical Papers (CHAMP). (DOCX)

S2 Fig Trajectory graphs for sensitivity analysis #4. Joint Space Width (JSW) trajectory groups across 96-months of follow-up among the *imputed cohort*. **Panel A** shows trajectories among previously injured male right knees from men (n = 383), while **Panel B** shows trajectories among previously injured right knees from women. JSW measurements were imputed using the “last observation carried forward method.” The red trajectories represent the “low baseline JSW” group, the green trajectories represent the “medium baseline JSW” group, and the blue trajectories represent the “high baseline JSW” group.
(PNG)
