# Supplementary material for: Identifying trajectories of joint space width loss among previously injured knees: Data from the Osteoarthritis Initiative
Source: PLoS One. 2025 Jun 30;20(6):e0325822. doi: 10.1371/journal.pone.0325822 (PMC12208416; doi:10.1371/journal.pone.0325822)
Supplement: S1 Table — Censored normal distribution model fitting statistics for group-based trajectory modeling among men (from the n = 1366 knee cohort). Models include time (independent variable) and joint space width (dependent variable). (DOCX) [file pone.0325822.s001.docx]

| **# Groups** | **Polynomial Order(s)** | **Term** | **Group 1**  *Beta* (SE)  *p-value* | **Group 2**  *Beta* (SE)  *p-value* | **Group 3**  *Beta* (SE)  *p-value* | **Group 4**  *Beta* (SE)  *p-value* | **BIC** |
| --- | --- | --- | --- | --- | --- | --- | --- |
| 1 | Quadratic | Intercept  Linear    Quadratic | 5.90 (0.14)  *P < 0.001*  -0.19 (0.09)  *P = 0.03*  0.02 (0.01)  *P = 0.09* |  |  |  | -5325.3 |
| 1 | Linear | Intercept  Linear | 5.70 (0.08)  *P < 0.001*  -0.05 (0.02)  *P = 0.01* |  |  |  | - 4519.05 |
| 2 | Linear  Linear | Intercept  Linear | 2.79 (0.15)  *P < 0.001*  -0.08 (0.04)  *P = 0.02* | 6.59 (0.06)  *P < 0.001*  -0.12 (0.01)  *P < 0.001* |  |  | - 4562.6 |
| 2 | Linear  Quadratic | Intercept  Linear  Quadratic | 3.38 (0.24)  *P < 0.001*  -0.51 (0.14)  *P < 0.001*  0.06 (0.02)  *P = 0.002* | 6.59 (0.06)  *P < 0.001*  -0.12 (0.01)  *P < 0.001* |  |  | - 4561.0 |
| 3 | Linear  Linear  Linear | Intercept  Linear | 2.01 (0.13)  *P < 0.001*  -0.10 (0.04)  *P = 0.004* | 5.53 (0.06)  *P < 0.001* |  |  |  |
| 3 | Quadratic  Linear  Linear | Intercept  Linear  Quadratic | 2.55 (0.21)  *P < 0.001*  -0.50 (0.13)  *P < 0.001*  0.06 (0.02)  *P = 0.002* | 5.53 (0.06)  *P < 0.001*  -0.14 (0.01)  *P < 0.001* | 7.51 (0.07)  *P < 0.001*  -0.11 (0.01)  *P < 0.001* |  | - 3889.9 |
| 4 | Quadratic  Linear  Linear  Linear | Intercept  Linear  Quadratic | 2.44 (0.18)  *P < 0.001*  -0.49 (0.12)  *P< 0.001*  0.06 (0.02)  *P < 0.001* | 5.19 (0.06)  *P < 0.001*  -0.17 (0.02)  *P < 0.001* | 6.55 (0.06)  *P < 0.001*  -0.12 (0.01)  *P < 0.001* | 8.03 (0.08)  *P < 0.001*  -0.09 (0.02)  *P < 0.001* | - 3578.8 |

Note: *Beta* (SE) = parameter estimate and associated standard error. *P-value* = suggest significance for each regression term. BIC = Bayes Information Criteria.
