# Supplementary material for: Identifying trajectories of joint space width loss among previously injured knees: Data from the Osteoarthritis Initiative
Source: PLoS One. 2025 Jun 30;20(6):e0325822. doi: 10.1371/journal.pone.0325822 (PMC12208416; doi:10.1371/journal.pone.0325822)
Supplement: S2 Table — Censored normal distribution model fitting statistics for group-based trajectory modeling among women (from the n = 1366 knee cohort). Models include time (independent variable) and joint space width (dependent variable). (DOCX) [file pone.0325822.s002.docx]

| **# Groups** | **Polynomial Order(s)** | **Term** | **Group 1**  *Beta* (SE)  *p-value* | **Group 2**  *Beta* (SE)  *p-value* | **Group 3**  *Beta* (SE)  *p-value* | **Group 4**  *Beta* (SE)  *p-value* | **BIC** |
| --- | --- | --- | --- | --- | --- | --- | --- |
| 1 | Quadratic | Intercept  Linear    Quadratic | 5.31 (0.11)  *P < 0.001*  -0.12 (0.06)  *P = 0.06*   - 1. (0.01)   *P = 0.40* |  |  |  | - 4678.8 |
| 1 | Linear | Intercept  Linear | 5.24 (0.06)  *P < 0.001*  -0.07 (0.02)  *P < 0.001* |  |  |  | - 4676.1 |
| 2 | Linear  Linear | Intercept  Linear | 3.83 (0.11)  *P < 0.001*  -0.10 (0.02)  *P < 0.001* | 5.98 (0.06)  *P < 0.001*  -0.09 (0.01)  *P < 0.001* |  |  | - 4011.5 |
| 2 | Linear  Quadratic | Intercept  Linear  Quadratic | 4.03 (0.15)  *P < 0.001*  -0.25 (0.08)  *P = 0.003*   - 1. (0.01)   *P = 0.06* | 5.98 (0.06)  *P < 0.001*  -0.09 (0.01)  *P < 0.001* |  |  | - 4012.9 |
| 3 | Linear  Linear  Linear | Intercept  Linear | 3.14 (0.10)  *P < 0.001*  -0.15 (0.02)  *P < 0.001* | 5.16 (0.05)  *P < 0.001*  -0.11 (0.01)  *P < 0.001* | 6.63 (0.06)  *P < 0.001*  -0.08 (0.01)  *P < 0.001* |  | - 3535.6 |
| 3 | Quadratic  Linear  Linear | Intercept  Linear  Quadratic | 3.39 (0.16)  *P < 0.001*  -0.35 (0.10)  *P < 0.001*   - 1. (0.01)   *P = 0.04* | 5.16 (0.05)  *P < 0.001*  -0.11 (0.01)  *P < 0.001* | 6.63 (0.06)  *P < 0.001*  -0.08 (0.01)  *P < 0.001* |  | - 3536.5 |
| 4 | Quadratic  Linear  Linear  Linear | Intercept  Linear  Quadratic | 3.03 (0.17)  *P < 0.001*  -0.50 (0.11)  *P < 0.001*   - 1. (0.02)   *P = 0.002* | 4.71 (0.06)  *P < 0.001*  -0.15 (0.01)  *P < 0.001* | 5.85 (0.06)  *P < 0.001*  -0.09 (0.01)  *P < 0.001* | 7.40 (0.13)  *P < 0.001*  -0.05 (0.02)  *P = 0.03* | -3232.7 |

Note: *Beta* (SE) = parameter estimate and associated standard error. *P-value* = suggest significance for each regression term. BIC = Bayes Information Criteria.
