# Supplementary material for: Identifying trajectories of joint space width loss among previously injured knees: Data from the Osteoarthritis Initiative
Source: PLoS One. 2025 Jun 30;20(6):e0325822. doi: 10.1371/journal.pone.0325822 (PMC12208416; doi:10.1371/journal.pone.0325822)
Supplement: S3 Table — Censored normal distribution group-based trajectory model fitting statistics for n = 383 right knees from men in the primary cohort. Models include time (independent variable) and joint space width (dependent variable). (DOCX) [file pone.0325822.s003.docx]

| **# Groups** | **Polynomial Order(s)** | **Term** | **Group 1**  *Beta* (SE)  *p-value* | **Group 2**  *Beta* (SE)  *p-value* | **Group 3**  *Beta* (SE)  *p-value* | **Group 4**  *Beta* (SE)  *p-value* | **BIC** |
| --- | --- | --- | --- | --- | --- | --- | --- |
| 1 | Quadratic | Intercept  Linear    Quadratic | 6.14 (0.14)  *P < 0.001*  -0.20 (0.08)  *P = 0.02*  0.02 (0.01)  *P = 0.13* |  |  |  | - 4520.9 |
| 1 | Linear | Intercept  Linear | 5.96 (0.08)  *P < 0.001*  -0.08 (0.02)  *P < 0.001* |  |  |  | - 4519.1 |
| 2 | Linear  Linear | Intercept  Linear | 4.6 (0.08)  *P < 0.001*  -0.11 (0.02)  *P < 0.001* | 7.12 (0.07)  *P < 0.001*  -0.11 (0.02)  *P < 0.001* |  |  | - 3828.4 |
| 2 | Linear  Quadratic | Intercept  Linear  Quadratic | 4.9 (0.14)  *P < 0.001*  -0.31 (0.08)  *P < 0.001*  0.03 (0.01)  *P = 0.02* | 7.17 (0.07)  *P < 0.001*  -0.11 (0.02)  *P < 0.01* |  |  | - 3828.4 |
| 3 | Linear  Linear  Linear | Intercept  Linear | 3.15 (0.12)  *P < 0.001*  -0.18 (0.03)  *P < 0.001* | 5.8 (0.06)  *P < 0.001*  -0.14 (0.02)  *P < 0.001* | 7.64 (0.07)  *P < 0.001*  -0.10 (0.02)  *P < 0.001* |  | - 3288.3 |
| **3** | **Quadratic**  **Linear**  **Linear** | **Intercept**  **Linear**  **Quadratic** | **3.60 (0.20)**  ***P < 0.001***  **-0.50 (0.12)**  ***P < 0.001***  **0.04 (0.01)**  ***P = 0.01*** | **5.80 (0.06)**  ***P < 0.001***  **-0.14 (0.01)**  ***P < 0.001*** | **7.64 (0.07)**  ***P < 0.001***  **-0.10 (0.02)**  ***P < 0.001*** |  | **- 3287.8** |
| 4 | Quadratic  Linear  Linear  Linear | Intercept  Linear  Quadratic | 3.26 (0.20)  *P < 0.001*  -0.55 (0.12)  *P < 0.001*  0.05 (0.02)  *P = 0.003* | 5.31 (0.06)  *P < 0.001*  -0.17 (0.01)  *P < 0.001* | 6.58 (0.06)  *P < 0.001*  -0.11 (0.01)  *P < 0.001* | 8.02 (0.07)  *P < 0.001*  -0.08 (0.02)  *P < 0.001* | - 2966.6 |

Note: Best fitting model highlighted in **bold**. *Beta* (SE) = parameter estimate and associated standard error. *P-value* = suggest significance for each regression term. BIC = Bayes Information Criteria.
