# Supplementary material for: Identifying trajectories of joint space width loss among previously injured knees: Data from the Osteoarthritis Initiative
Source: PLoS One. 2025 Jun 30;20(6):e0325822. doi: 10.1371/journal.pone.0325822 (PMC12208416; doi:10.1371/journal.pone.0325822)
Supplement: S4 Table — Censored normal distribution group-based trajectory model fitting statistics for n = 389 right knees from women in the primary cohort. Models include time (independent variable) and joint space width (dependent variable). (DOCX) [file pone.0325822.s004.docx]

| **# Groups** | **Polynomial Order(s)** | **Term** | **Group 1**  β (SE)  *p-value* | **Group 2**  β (SE)  *p-value* | **Group 3**  β (SE)  *p-value* | **Group 4**  β (SE)  *p-value* | **BIC** |
| --- | --- | --- | --- | --- | --- | --- | --- |
| 1 | Quadratic | Intercept  Linear    Quadratic | 5.43 (0.11)  *P < 0.001*  -0.16 (0.07)  *P = 0.02*   - 1. (0.01)   *P = 0.23* |  |  |  | - 4069.1 |
| 1 | Linear | Intercept  Linear | 5.32 (0.06)  *P < 0.001*  -0.08 (0.01)  *P < 0.001* |  |  |  | - 4066.9 |
| 2 | Linear  Linear | Intercept  Linear | 4.05 (0.08)  *P < 0.001*  -0.10 (0.02)  *P < 0.001* | 6.06 (0.06)  *P < 0.001*  -0.09 (0.01)  *P < 0.001* |  |  | - 3466.2 |
| 2 | Linear  Quadratic | Intercept  Linear  Quadratic | 4.27 (0.14)  *P < 0.001*  -0.25 (0.08)  *P = 0.002*   - 1. (0.01)   *P = 0.06* | 6.06 (0.06)  *P < 0.001*  -0.09 (0.01)  *P < 0.001* |  |  | - 3467.4 |
| **3** | **Linear**  **Linear**  **Linear** | **Intercept**  **Linear** | **3.30 (0.10)**  ***P < 0.001***  **-0.16 (0.02)**  ***P < 0.001*** | **5.21 (0.05)**  ***P < 0.001***  **-0.11 (0.01)**  ***P < 0.001*** | **6.64 (0.06)**  ***P < 0.001***  **-0.08 (0.01)**  ***P < 0.001*** |  | **- 3027.4** |
| 3 | Quadratic  Linear  Linear | Intercept  Linear  Quadratic | 3.50 (0.17)  *P < 0.001*  -0.30 (0.10)  *P = 0.003*   - 1. (0.01)   *P = 0.16* | 5.21 (0.05)  *P < 0.001*  -0.11 (0.01)  *P < 0.001* | 6.64 (0.06)  *P < 0.001*  -0.08 (0.01)  *P < 0.001* |  | - 3029.4 |

Note: Best fitting model highlighted in **bold**. *Beta* (SE) = parameter estimate and associated standard error. *P-value* = suggest significance for each regression term. BIC = Bayes Information Criteria.
