# Supplementary material for: Identifying trajectories of joint space width loss among previously injured knees: Data from the Osteoarthritis Initiative
Source: PLoS One. 2025 Jun 30;20(6):e0325822. doi: 10.1371/journal.pone.0325822 (PMC12208416; doi:10.1371/journal.pone.0325822)
Supplement: S5 Table — Censored normal distribution group-based trajectory model fitting statistics for n = 107 right knees from men in the subset cohort. Models include time (independent variable) and joint space width (dependent variable). (DOCX) [file pone.0325822.s005.docx]

| **# Groups** | **Polynomial Order(s)** | **Term** | **Group 1**  *Beta* (SE)  *p-value* | **Group 2**  *Beta* (SE)  *p-value* | **Group 3**  *Beta* (SE)  *p-value* | **Group 4**  *Beta* (SE)  *p-value* | **BIC** |
| --- | --- | --- | --- | --- | --- | --- | --- |
| 1 | Quadratic | Intercept  Linear    Quadratic | 6.17  *P = 0.00*  -0.29  *P = 0.03*  0.02  *P = 0.13* |  |  |  | -1200.28 |
| 1 | Linear | Intercept  Linear | 6.03  *P = 0.00*  -0.07  *P = 0.01* |  |  |  | -2712.05 |
| 2 | Linear  Linear | Intercept  Linear | 5.02  *P = 0.00*  -0.17  *P = 0.00* | 6.90  *P = 0.00*  -0.08  *P = 0.00* |  |  | -1002.75 |
| 2 | Linear  Quadratic | Intercept  Linear  Quadratic | 5.20  *P = 0.00*  -0.29  *P = 0.02*  0.02  *P = 0.30* | 6.91  *P = 0.00*  -0.08  *P =0.00* |  |  | -1004.56 |
| 2 | Quadratic  Quadratic | Intercept  Linear    Quadratic | 5.20  *P = 0.00*  -0.29  *P = 0.02*  0.02  *P = 0.30* | 6.98  *P = 0.00*  -0.13  *P =0.28*  0.01  *P = 0.68* |  |  | -1006.81 |
| **3** | **Linear**  **Linear**  **Linear** | **Intercept**  **Linear** | **3.88**  ***P=0.00***  **-0.23**  ***P = 0.00*** | **5.77**  ***P = 0.00***  **-0.19**  ***P = 0.00*** | **7.09**  ***P = 0.00***  **-0.07**  ***P = 0.00*** |  | **-892.97** |
| 4 | Linear  Linear  Linear  Linear | Intercept  Linear | 3.35  *P = 0.00*  -0.30  *P = 0.00* | 5.20  *P = 0.00*  -0.21  *P = 0.00* | 6.32  *P = 0.00*  -0.16  *P = 0.00* | 7.31  *P = 0.00*  -0.04  *P = 0.08* | -827.61 |
