# Supplementary material for: Identifying trajectories of joint space width loss among previously injured knees: Data from the Osteoarthritis Initiative
Source: PLoS One. 2025 Jun 30;20(6):e0325822. doi: 10.1371/journal.pone.0325822 (PMC12208416; doi:10.1371/journal.pone.0325822)
Supplement: S6 Table — Censored normal distribution group-based trajectory model fitting statistics for n = 144 right knees from women in the subset cohort. Models include time (independent variable) and joint space width (dependent variable). (DOCX) [file pone.0325822.s006.docx]

| **# Groups** | **Polynomial Order(s)** | **Term** | **Group 1**  *Beta* (SE)  *p-value* | **Group 2**  *Beta* (SE)  *p-value* | **Group 3**  *Beta* (SE)  *p-value* | **Group 4**  *Beta* (SE)  *p-value* | **BIC** |
| --- | --- | --- | --- | --- | --- | --- | --- |
| 1 | Quadratic | Intercept  Linear    Quadratic | 5.31  *P = 0.00*  -0.21  *P = 0.04*  0.02  *P = 0.23* |  |  |  | -1449.41 |
| 1 | Linear | Intercept  Linear | 5.14  *P = 0.00*  -0.09  *P = 0.00* |  |  |  | -1447.66 |
| 2 | Linear  Linear | Intercept  Linear | 3.90  *P = 0.00*  -0.13  *P = 0.00* | 5.83  *P = 0.00*  -0.10  *P = 0.00* |  |  | -1197.19 |
| 2 | Linear  Quadratic | Intercept  Linear  Quadratic | 4.90  *P = 0.00*  -0.20  *P = 0.17*  0.02  *P = 0.17* | 6.63  *P = 0.00*  -0.07  *P = 0.01* |  |  | -1577.97 |
| 2 | Quadratic  Quadratic | Intercept  Linear    Quadratic | 4.14  *P = 0.00*  -0.29  *P = 0.02*  0.02  *P = 0.16* | 5.83  *P = 0.02*  -0.10  *P = 0.22*  0.00  *P = 0.99* |  |  | -1201.14 |
| **3** | **Linear**  **Linear**  **Linear** | **Intercept**  **Linear** | **3.33**  ***P = 0.00***  **-0.13**  ***P = 0.00*** | **5.10**  ***P = 0.00***  **-0.12**  ***P = 0.00*** | **6.38**  ***P = 0.00***  **-0.10**  ***P = 0.00*** |  | **-1076.27** |
| 4 | Linear  Linear  Linear  Linear | Intercept  Linear | 2.68  *P = 0.00*  -0.23  *P = 0.00* | 4.52  *P = 0.00*  -0.20  *P = 0.00* | 5.32  *P = 0.00*  -0.09  *P = 0.00* | 6.52  *P = 0.00*  -0.11  *P = 0.00* | -948.61 |
