# Supplementary material for: Identifying trajectories of joint space width loss among previously injured knees: Data from the Osteoarthritis Initiative
Source: PLoS One. 2025 Jun 30;20(6):e0325822. doi: 10.1371/journal.pone.0325822 (PMC12208416; doi:10.1371/journal.pone.0325822)
Supplement: S8 Table — Censored normal distribution group-based trajectory model fitting statistics for n = 389 right knees from women in the imputed cohort. JSW measurements were imputed using the “last observation carried forward” method. Models include time (independent variable) and joint space width (dependent variable). (DOCX) [file pone.0325822.s008.docx]

| **# Groups** | **Polynomial Order(s)** | **Term** | **Group 1**  *Beta* (SE)  *p-value* | **Group 2**  *Beta* (SE)  *p-value* | **Group 3**  *Beta* (SE)  *p-value* | **Group 4**  *Beta* (SE)  *p-value* | **BIC** |
| --- | --- | --- | --- | --- | --- | --- | --- |
| 1 | Quadratic | Intercept  Linear    Quadratic | 5.42 (0.11)  *P = 0.00*  -0.14 (0.07)  *P = 0.04*  0.00 (0.01)  *P = 0.57* |  |  |  | -4878.23 |
| 1 | Linear | Intercept  Linear | 5.36 (0.06)  *P = 0.00*  -0.10 (0.01)  *P = 0.00)* |  |  |  | -4874.44 |
| 2 | Linear  Linear | Intercept  Linear | 3.91 (0.14)  *P = 0.00*  -0.17 (0.02)  *P = 0.00* | 5.88 (0.07)  *P = 0.00*  -0.07 (0.01)  *P = 0.00* |  |  | -4094.76 |
| 2 | Linear  Quadratic | Intercept  Linear  Quadratic | 3.91 (0.14)  *P = 0.00*  -0.17 (0.02)  *P = 0.00* | 5.90 (0.10)  *P = 0.00*  -0.09 (0.05)  *P = 0.10*  0.00 (0.01)  *P = 0.82* |  |  | -4098.69 |
| 3 | Linear  Linear  Linear | Intercept  Linear | 3.28 (0.09)  *P = 0.00*  -0.19 (0.02)  *P = 0.00* | 5.19 (0.05)  *P = 0.00*  -0.10 (0.01)  *P = 0.00* | 6.59 (0.06)  *P = 0.00*  -0.05 (0.01)  *P = 0.00* |  | -3517.26 |
| 4 | Linear  Linear  Linear  Linear | Intercept  Linear | 3.12 (0.09)  *P = 0.00*  -0.20 (0.02)  *P = 0.00* | 4.90 (0.06)  *P = 0.00*  -0.13 (0.01)  *P = 0.00* | 5.94 (0.05)  *P = 0.00*  -0.07 (0.01)  *P = 0.00* | 7.60 (0.10)  *P = 0.00*  -0.01 (0.02)  *P = 0.52* | -3128.6 |
