# Supplementary figures and images for: Identifying trajectories of joint space width loss among previously injured knees: Data from the Osteoarthritis Initiative
Source: PLoS One. 2025 Jun 30;20(6):e0325822. doi: 10.1371/journal.pone.0325822 (PMC12208416; doi:10.1371/journal.pone.0325822)

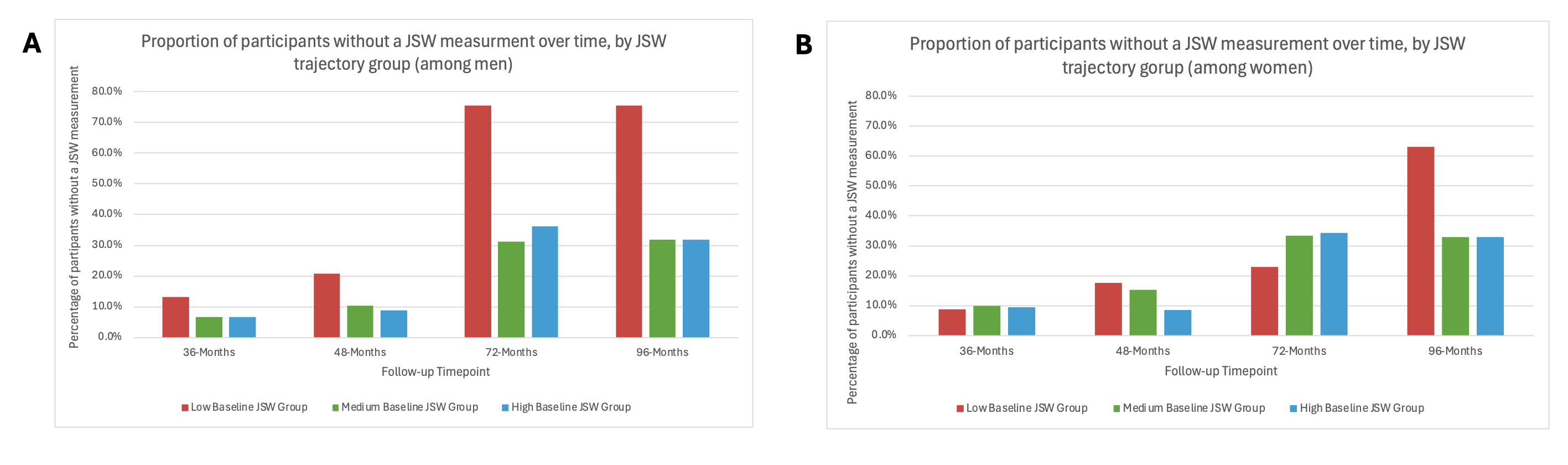

Supplement: S1 Fig — Frequencies of missing JSW observations at the 36-month, 48-month, 72-month, and 96-month follow-ups stratified by assigned trajectory groups among the primary cohort. The left-hand distribution includes knees from men, while the right-hand distribution includes knees from women. (PNG) [file pone.0325822.s009.png]

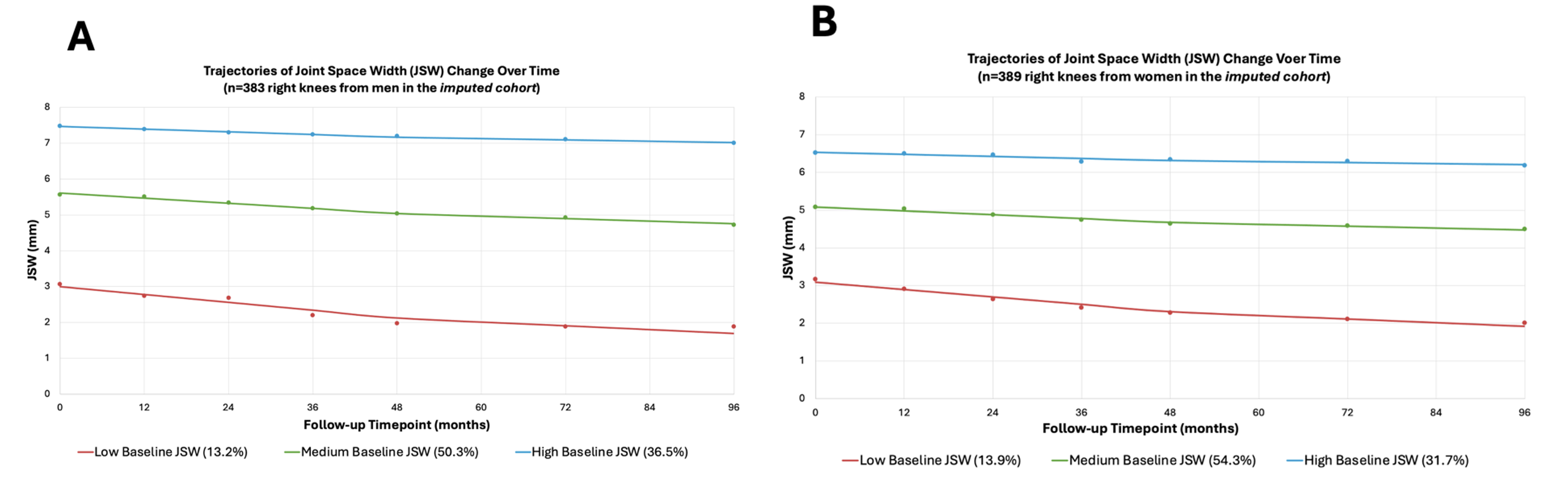

Supplement: S2 Fig — Joint Space Width (JSW) trajectory groups across 96-months of follow-up among the imputed cohort. Panel A shows trajectories among previously injured male right knees from men (n = 383), while Panel B shows trajectories among previously injured right knees from women. JSW measurements were imputed using the “last observation carried forward method.” The red trajectories represent the “low baseline JSW” group, the green trajectories represent the “medium baseline JSW” group, and the blue trajectories represent the “high baseline JSW” group. (PNG) [file pone.0325822.s011.png]
