# Supplementary material for: Identifying trajectories of joint space width loss among previously injured knees: Data from the Osteoarthritis Initiative
Source: PLoS One. 2025 Jun 30;20(6):e0325822. doi: 10.1371/journal.pone.0325822 (PMC12208416; doi:10.1371/journal.pone.0325822)
Supplement: S9 Table — (DOCX) [file pone.0325822.s010.docx]

| **Design and conduct** | | | | |
| --- | --- | --- | --- | --- |
| Clear description of the goal of research, study objective(s), study design, and study population | **Yes** | Unclear | No | N/A |
| Clear description of outcomes, exposures/treatments and covariates, and their measurement methods | **Yes** | Unclear | No | N/A |
| Validity of study design | **Yes** | Unclear | No | N/A |
| Clear statement and justification of sample size | **Yes** | Unclear | No | N/A |
| Clear declaration of design violations and acceptability of the design violations | **Yes** | Unclear | No | N/A |
| Consistency between the paper and its previously published protocol | **Yes** | Unclear | No | N/A |
| **Data analysis** | | | | |
| Correct and complete description of statistical methods | **Yes** | Unclear | No | N/A |
| Valid statistical methods used and assumptions outlined | **Yes** | Unclear | No | N/A |
| Appropriate assessment of treatment effect or interaction between treatment and another covariate | **Yes** | Unclear | No | N/A |
| Correct use of correlation and associational statistical testing | **Yes** | Unclear | No | N/A |
| Avoiding model extrapolation not supported by data | **Yes** | Unclear | No | N/A |
| Adequate handling of missing data | **Yes** | Unclear | No | N/A |
| **Reporting and presentation** | | | | |
| Adequate and correct description of the data | **Yes** | Unclear | No | N/A |
| Descriptive results provided as occurrence measures with confidence intervals, and analytic results provided as association measures and confidence intervals along with p-values | **Yes** | Unclear | No | N/A |
| Confidence intervals provided for the contrast between groups rather than for each group | Yes | Unclear | No | **N/A** |
| Avoiding selective reporting of analyses and p-hacking | Yes | Unclear | No | **N/A** |
| Providing sufficient numerical results that could be included in a subsequent meta-analysis | **Yes** | Unclear | No | N/A |
| Acceptable presentation of the figures and tables | **Yes** | Unclear | No | N/A |
| **Interpretation** | | | | |
| Interpreting the results based on association measures and 95% confidence intervals along with p-values, and correctly interpreting large p-values as indecisive results, not evidence of absence of an effect | Yes | Unclear | No | **N/A** |
| Using confidence intervals rather than post-hoc power analysis for interpreting the results of studies | Yes | Unclear | No | **N/A** |
| Correctly interpreting occurrence or association measures | **Yes** | Unclear | No | N/A |
| Distinguishing causation from association and correlation | **Yes** | Unclear | No | N/A |
| Results of pre-specified analyses are distinguished from the results of exploratory analyses in the interpretation | **Yes** | Unclear | No | N/A |
| Appropriate discussion of the study methodological limitations | **Yes** | Unclear | No | N/A |
| Drawing only conclusions supported by the statistical analysis and no generalization of the results to subjects outside the target population | **Yes** | Unclear | No | N/A |
